# Editorial: Artificial intelligence and multimodal medical imaging data fusion for improving cardiovascular disease care

**DOI:** 10.3389/fradi.2024.1412404

**Published:** 2024-11-15

**Authors:** Saeed Amal, Douglas Sawyer, Arda Könik

**Affiliations:** ^1^ The Roux Institute at Northeastern University, Portland, ME, United States; ^2^ Department of Bioengineering, Northeastern University, Boston, MA, United States; ^3^ Maine Medical Center, MaineHealth, Portland, ME, United States; ^4^ Department of Imaging, Dana–Farber Cancer Institute, Boston, MA, United States

**Keywords:** artificial intelligence, multimodal medical data fusion, cardiovascular disease, multimodal machine learning, multimodal deep learning, machine learning (ML), machine learning and AI

**Editorial on the Research Topic**
Artificial intelligence and multimodal medical imaging data fusion for improving cardiovascular disease care

## Introduction

Today's digital health aims to provide an improved efficiency of healthcare delivery, and personalized, and timely disease care. Cardiovascular Disease (CVD) is a leading cause of death worldwide. In the United States, 1 out of 3 adults have some form of CVD. It is projected that nearly half of the US population will have at least one type of CVD by 2035, with total direct and indirect costs potentially surpassing $1 trillion (1–3). Medical imaging data encompass multiple modalities that are primarily utilized in silos. These include Computed Tomography (CT), Magnetic Resonance Imaging (MRI), CT-derived fractional flow reserve (CT-FFR), cardiac MRI, whole-heart dynamic 3D cardiac MRI perfusion, 3D cardiac MRI late gadolinium enhancement, cardiac positron emission tomography (PET), echocardiography and coronary angiography. However, only a few modalities are utilized in hybrid configurations, such as Positron Emission Tomography combined with Computed Tomography (PET/CT),Single-Photon Emission Computed Tomography combined with CT (SPECT/CT), Echocardiography and invasive angiography. Integrating these different imaging modalities becomes a burden on clinicians as it can lead to added complexity, potential inaccuracies, and increased healthcare costs. This research topic focused on fusion techniques that enable the integration and modeling of these multiple modalities to offer complementary information that will help improve CVD care. These modalities will leverage Machine Learning (ML) and Deep Learning (DL) techniques as well as other state-of-the-art techniques. Following are some insights and findings from this research topic:

Milosevic et al. conducted a systematic and comprehensive review on the state-of-the-art multi-modal medical data fusion in the context of CVD (Milosevic et al.). Their review indicated that there are limited open multimodal datasets that are constrained both in size and modality scope. This scarcity of open datasets of labeled pathologies contributes to the comparatively few published papers on the diagnosis or prediction of cardiovascular diseases and conditions. The review indicated that over the last 5 years, there has been a considerable amount of work in artificial intelligence employing fusion techniques of multi-modal imaging involving various magnetic resonance imaging (MRI) and CT scans. However, the integration of modalities like x-ray, echocardiography, and non-imaging modalities remains relatively scarce.

Yang et al. compared the performance of a deep learning (DL)-based automatic left ventricle (LV) segmentation network trained primarily on CT images in two whole-heart cardiac magnetic resonance (CMR) reconstruction methods: (1) an in-line respiratory motion-corrected (Mcorr) reconstruction and (2) an off-line, compressed sensing-based, Mres reconstruction (Yang et al.). They found that the DL-based 3D automatic LV segmentation network trained on CT images and fine-tuned on MR images generalized better on Mres images than on Mcorr images for quantifying LV volumes.

Penso et al. evaluated whether a DL model could identify myocardial fibrosis from routine early contrast-enhanced cardiac CT (CE-CCT) images (Penso et al.). The authors found that DL on early CE-CCT acquisition may allow detection of LV sectors affected with myocardial fibrosis without the need for additional contrast-agent administration or radiation dose. Furthermore, such a tool might reduce the user interaction and visual inspection, saving both effort and time.

Nguyen et al. investigated the utility of high-dimensional longitudinal imaging data of five modalities, both individually and collectively, for dynamic prediction of cardiovascular and renal disease (CVRD) in young adults in a multi-centered cohort followed up over 30 years (Nguyen et al.). The authors used the entirety of imaging variables over all exam years for continuously updated predictions of risk and found that longitudinal imaging data, even when irregularly collected and featuring high rates of missing data, improved CVRD dynamic prediction (3% iAUC, up to 5% C-index in midlife).

### Summary

Effective use of AI to interpret multimodal medical data for cardiovascular disease has the potential to significantly impact our understanding of an individual's cardiovascular function and disease burden. There has been significant scholarship on fusion and segmentation of multimodal imaging, with significant improvements in performance compared to single modality modals. While multimodal modals show significant promise,there is considerable work needed to further develop AI as a valuable tool in the hands of well-trained clinicians in regards to developing diagnosis and prediction models and with integrating a wider variety of imaging modalities. We are excited to see this work advance, which will lead to enhancements in clinical decision-making, improvements in patient outcomes and reductions in healthcare costs.
